# An Integrated Experimental System for Unmanned Underwater Vehicle Swarm Control

**DOI:** 10.3390/s25206413

**Published:** 2025-10-17

**Authors:** Yutao Chen, Xingwei Zhou, Wenshan Hu, Bo Zhao

**Affiliations:** 1 Naval University of Engineering, Wuhan 430030, China; 0908041018@nue.edu.cn (Y.C.); 1920191099@nue.edu.cn (B.Z.); 2 Wuhan University, Wuhan 430072, China; wenshan.hu@whu.edu.cn

**Keywords:** unmanned underwater vehicle swarm control, integrated experimental platform, rapid prototyping and simulation, digital twin system

## Abstract

Unmanned Underwater Vehicle (UUV) swarms have become increasingly crucial for underwater exploration and applications, where their coordinated operation offers significant advantages over single-vehicle systems. However, unlike single-vehicle systems, the development of swarm control systems is more complicated, especially because there are limited integrated toolchains that can cover both global scheme design and individual vehicle implementation. Engineers may have to develop a global scheme and then partition it manually for individual vehicle implementation, which can result in substantial efficiency losses. To address this difficulty, an integrated experimental framework is developed to support the complete workflow of UUV swarm control development, from unified algorithm design and system simulation to automated code generation and individual deployment. The architecture of the proposed platform incorporates three principal elements: a global simulation environment that enables virtual validation of swarm collective behavior, a rapid prototyping module that facilitates code generation/partitioning and individual implementation, and a digital twin visualization component that provides real-time monitoring capabilities. A case study demonstrates that the platform can integrate global design with individual implementation. In a comparative experiment where the same engineering team implemented a three-UUV formation control algorithm, the use of our platform reduced the time from algorithm design to successful deployment from an estimated 6 h (using manual coding and integration) to under one hour, representing about an 80% reduction in development time.

## 1. Introduction

Unmanned Underwater Vehicle (UUV) swarm control plays a pivotal role in underwater exploration, cooperative operation, and ocean resource exploration, offering significant potential to enhance maritime surveillance and strengthen underwater exploration capabilities beyond single-vehicle systems [1,2]. The development of UUV swarm systems aligns with the evolving demands of modern undersea exploration [3,4]. However, the transition from theoretical swarm control algorithms to reliably deployed multi-vehicle systems presents substantial challenges. Traditional development processes are often fragmented: engineers first design a global control scheme in high-level simulation environments and then manually partition, translate, and implement the algorithm into executable code for each individual vehicle. This manual process is not only time-consuming and labor-intensive but also prone to errors, leading to a significant “sim-to-real” performance gap and hindering rapid prototyping.

While numerous UUV swarm control algorithms have been proposed in recent literature, a majority of these studies remain confined to simulation-based validation within generic environments, lacking dedicated experimental platforms that bridge the gap to real-world verification and deployment [5]. Existing platforms for multi-agent systems, whether virtual, physical, or hybrid, involve inherent trade-offs in experimental flexibility, scalability, and translational efficiency to physical hardware. This gap in the development ecosystem underscores the need for an integrated framework that seamlessly supports the complete workflow from unified algorithm design and system-wide simulation to automated code generation and individual deployment.

To address these limitations, this paper proposes an integrated experimental platform for UUV swarm control. The primary objective is to support the entire development lifecycle within a unified environment, thereby streamlining the transition from conceptual algorithm design to operational swarm deployment. The key contributions of this work are as follows:

1. It introduces a Browser/Server (B/S) architecture-based platform that integrates global swarm algorithm design, system-wide co-simulation, automated code generation, and distributed deployment into a cohesive workflow.
2. It features a self-developed graphical programming and compilation engine that eliminates reliance on commercial tools like MATLAB/Simulink, automatically translating control block diagrams into executable code for distributed controllers.
3. It incorporates a digital twin visualization component, providing real-time 3D monitoring and facilitating dynamic parameter tuning during both simulation and physical operation.
4. The platform’s efficacy is validated through a case study involving a leader–follower formation control of multiple UUVs, demonstrating a significant reduction in development time compared to conventional manual approaches.

The remainder of this paper is organized as follows. Section 2 provides a comprehensive review of related work. Section 3 details the overall architecture of the proposed experimental system. Section 4 elaborates on the implementation of its core capabilities. Section 5 describes the experimental setup, including the hardware/software configuration and system parameters. Section 6 presents the experimental case study and results. Section 7 provides a comprehensive performance and scalability analysis of the platform, featuring quantitative metrics and stress tests. Finally, Section 8 concludes the paper and outlines specific directions for future research.

## 2. Related Works

The research on UUV swarm control is built upon the broader field of multi-agent systems. Despite the proliferation of control algorithms in recent literature, many studies focus on theoretical design and validation within generic simulation environments. For instance, references such as [6,7] primarily emphasize algorithm design and validate their methods using simulations in MATLAB or Unity, without conducting verification on a practical swarm control platform [5]. This highlights a common gap between algorithmic innovation and practical implementation.

Platforms for developing and testing multi-agent systems can be broadly categorized into three types. Fully virtual platforms, such as Gazebo [8,9], Webots [10,11], and CoppeliaSim [12,13], provide low-cost, accessible environments by simulating both agents and their environment, eliminating the need for physical hardware. In contrast, physical testbeds like Robotarium [14] and the Pi-puck ecosystem [15] enable algorithm validation on real-world multi-robot systems, offering high fidelity but often at the cost of reduced flexibility and higher maintenance. Hybrid platforms combine virtual and physical agents, exemplified by digital twin systems where virtual agents mirror and interact with their physical counterparts in real time [16]. This concept is gaining traction in underwater applications, with recent efforts like DARPA-supported tests using REMUS-class UUVs validating dual digital twin architectures for mission planning under constrained communication [17,18].

Each of these platform classifications presents inherent trade-offs in terms of experimental flexibility, scalability, and operational constraints [19]. Model-driven design frameworks like CPSwarm [20] have inspired streamlined, end-to-end workflows for cyber-physical swarms by demonstrating efficient transitions from design to hardware execution. However, a review of existing solutions reveals a persistent lack of a dedicated, integrated platform that seamlessly combines a unified design environment, system-level simulation, automated code generation for distributed UUV controllers, and integrated digital twin visualization within a single, cohesive framework.

A comparative analysis of our platform against representative existing platforms is summarized in Table 1, highlighting the integrated capabilities that distinguish our work. While existing platforms excel in specific areas such as pure simulation (Gazebo, Webots) or physical robot testing (Robotarium), they often lack a seamless, end-to-end workflow from unified algorithm design to automated deployment for underwater swarm systems.

As illustrated in Table 1, the proposed platform is uniquely characterized by its integration of a unified graphical design environment, UUV-specific modeling, automated code generation for distributed systems, and digital twin visualization into a single, cohesive end-to-end workflow. This integration directly addresses the fragmentation issue prevalent in current methodologies, where researchers often need to chain multiple disparate tools together. Unlike Robotarium, which provides remote access to physical robots but with fixed hardware and pre-defined interfaces, our platform allows for custom algorithm deployment on user-specified hardware. Furthermore, while a framework like CPSwarm shares the model-driven philosophy for swarm design, our implementation is specifically tailored for UUV applications, incorporating domain-specific dynamics and a robust digital twin system for validation and monitoring. This combination of features provides a comprehensive solution that significantly accelerates the UUV swarm development cycle.

The motivation for this work stems directly from the identified gaps in the current ecosystem. The absence of such an integrated toolchain forces researchers and engineers to juggle multiple disparate tools, leading to inefficient, error-prone, and protracted development cycles for UUV swarm systems. Our work aims to bridge this critical gap by providing a unified platform that supports the entire development workflow, thereby significantly accelerating the transition from theoretical algorithms to operational underwater swarm systems.

## 3. Overall Structure of the UUV Experimental System

**Figure 1:**
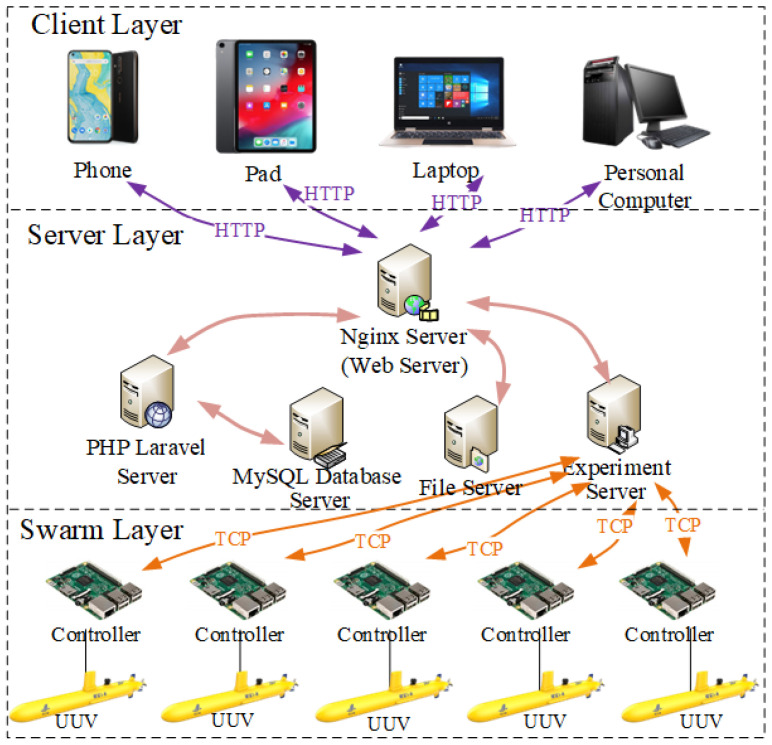
Overall structure of the UUV experimental system.

The UUV experimental system employs a Browser/Server (B/S) architecture, which offers significant advantages including cross-platform compatibility, low maintenance requirements, and excellent scalability [21]. The proposed architecture adopts a three-tiered design comprising the client layer, server layer, and swarm layer, which is illustrated in Figure 1. The client layer serves as the user interface, providing browser-based access to system functionalities. At the core of the architecture, the server layer handles critical business logic processing while managing bidirectional communication between clients and swarms. This intermediate layer processes user requests from clients and coordinates with the underlying swarm layer, which comprises the controllers and their associated UUVs. The swarm layer executes control algorithms and returns signal data through the server layer back to client interfaces. This layered approach ensures proper separation of concerns, which maintains efficient data flow throughout the system.

### 3.1. Client Layer

The client layer is implemented using the React framework with Ant-design components for user interface construction. For three-dimensional visualization capabilities, the system integrates both three.js and Unity rendering engines to handle complex animation requirements. State management is efficiently handled through a Reflux architecture, which minimizes unnecessary component rendering by maintaining a unidirectional data flow. Communication with the server layer is established through a combination of HTTP for RESTful API interactions and WebSocket for real-time data streaming. The frontend application is bundled with Webpack, and client-side routing is managed by React Router to enable seamless page transitions. Additionally, real-time experimental data visualization is achieved using Echarts, providing dynamic graphical representations of system status and performance metrics.

### 3.2. Server Layer

The server layer constitutes the core computational backbone of the system, responsible for executing critical business logic and coordinating data flow. Architecturally, this layer employs a modular service design comprising several specialized server components: an Nginx reverse proxy server that handles all incoming requests and load balancing; a PHP Laravel application server that processes RESTful API calls and manages business logic; an experimental management server dedicated to code generation, simulation execution, and experimental data transmission; a MYSQL database server that persistently stores system data including user profiles, algorithm block diagrams, monitoring configurations, and algorithm libraries; finally, a file server that hosts both compiled frontend assets and automatically generated algorithm executables. This distributed architecture enables each server component to optimize its designed functionality while maintaining efficient inter-server communication through well-defined interfaces. The separation of concerns across specialized servers enhances system reliability, scalability, and maintainability, with the Nginx server serving as the unified gateway that assigns request routing across all backend services.

### 3.3. Swarm Layer

The swarm layer represents the physical implementation tier of the system architecture, comprising two fundamental components: control units (controllers) and actuation elements (controlled objects). The control units serve as embedded execution systems that receive compiled algorithms via the Socket protocol and subsequently implement the prescribed control logic. These controllers maintain bidirectional real-time communication with the server layer through WebSocket connections, enabling continuous transmission of experimental data. Actuation elements can be either physical devices or virtual models. Physical actuation components interface with controllers through direct electrical connections, executing control actions on the UUV platform. Virtual actuation elements are mathematical representations embedded within the control algorithms themselves, simulating system dynamics and responses without physical instantiation. Notably, this architecture inherently supports hardware-in-the-loop (HIL) testing, where physical controllers interact with simulated plant models in real time, enabling rigorous validation of control strategies before field deployment. This dual approach enhances system flexibility by applying the same control methods to both simulations and real-world tests.

This section has presented the comprehensive architecture of the UUV experimental system, which adopts a three-layer B/S design for enhanced flexibility and scalability. The layered architecture achieves effective separation of concerns while ensuring reliable data flow throughout the system, supporting both simulation and physical testing scenarios through consistent control methodologies.

## 4. Implementation of System-Wide Simulation, Code Generation, and Deployment

**Figure 2:**
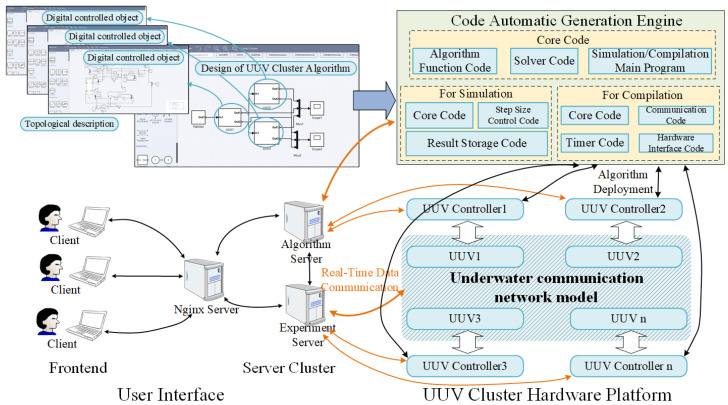
The integrated experimental system structure diagram of the control system for the UUV cluster.

The proposed experimental system demonstrates three fundamental characteristics: (i) system-wide cosimulation capability, (ii) automated code generation, and (iii) distributed deployment functionality. The UUV swarm framework proposed in this paper (shown in Figure 2) overcomes existing methodological limitations by integrating these features into a unified workflow for coordinated control algorithm development. Unlike conventional segmented approaches, the proposed framework provides one unified environment where users can create control algorithms, whether for individual UUVs or for coordinating the whole group together. The integrated simulation environment tests component interactions and control logic before implementation. The integrated environment then automatically generates executable code from these validated models, maintaining consistent performance between simulation and real deployment.

The following subsections elaborate on the key characteristics of the proposed platform.

### 4.1. Automated Code Generation

The coordinated control platform presented in this paper implements an innovative graphical algorithm design framework, similar to Simulink yet independently developed. This architecture establishes automated code generation as the foundational capability enabling both system-level cosimulation and distributed deployment. The system features a unified client interface for comprehensive swarm algorithm development, where individual controller algorithms are modularized as interconnected subsystems within the complete swarm control scheme. Notably, the simulation environment processes the entire swarm algorithm as an integrated control system, while the compilation phase automatically decomposes it into discrete subsystem executables. Distinct from conventional approaches utilizing MATLAB/Simulink, the solution in this paper designs a code generation engine specifically optimized for swarm control applications, offering enhanced customization and performance for distributed UUV systems.

**Figure 3:**
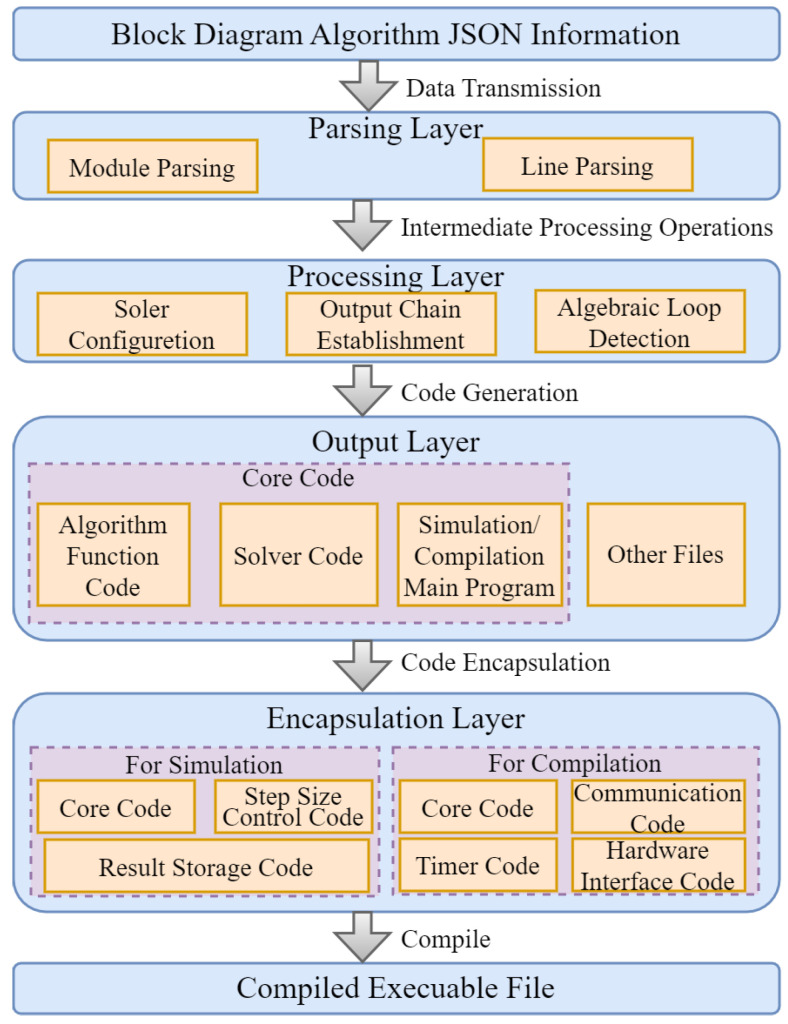
Algorithm to automatically generate flowcharts.

This section explains the operational workflow and underlying principles of the system. The graphical programming interface maintains a one-to-one correspondence between blocks in the frontend and their underlying code blocks stored on the server. The system automatically assembles these code blocks according to the connection relationships set in the graphical diagram, in which case, the complete project is integrated and then undergoes compilation to generate executable control programs [22]. The specific process can be divided into parsing layer, processing layer, output layer, and encapsulation layer, which is shown in Figure 3.

#### 4.1.1. Parsing Layer

This layer is responsible for reading and parsing the JSON data transmitted from the frontend, extracting critical information including module types, connection relationships, and module parameters. This layer produces preliminary module descriptions for subsequent automated code generation.

#### 4.1.2. Processing Layer

The intermediate processing steps involve the following: (i) a solver configuration to incorporate appropriate numerical algorithms during code generation, (ii) output link construction to ensure proper execution of the block diagram algorithm, and (iii) algebraic loop detection to prevent potential deadlock situations in the algorithmic implementation.

#### 4.1.3. Output Layer

Based on the output links generated by the processing layer, this layer invokes predefined code templates to generate corresponding code blocks for each module. These code blocks typically include initialization functions, update functions, and output functions. The primary responsibility of this layer is comprehensive code generation, producing the main program, solver code, algorithmic function code, and other relevant files.

#### 4.1.4. Encapsulation Layer

During the compilation process, this layer encapsulates core control code, communication protocols, timer code, etc., to form a complete project. The system ultimately compiles these components to generate executable binaries.

In the implementation, each block diagram module in the front-end corresponds to a well-defined code block on the server side. These code blocks are composed of the core functions mentioned earlier and are assembled in the processing layer according to predefined output links.

The construction of output links is a critical step in automatic code generation, as it determines the sequence in which the module code blocks are combined. If the order of the output links is incorrect, the logic of the generated control code will be flawed, leading to system malfunctions. Therefore, the accuracy of output links is essential to ensuring the reliability of the automatic code generation process.

**Figure 4:**
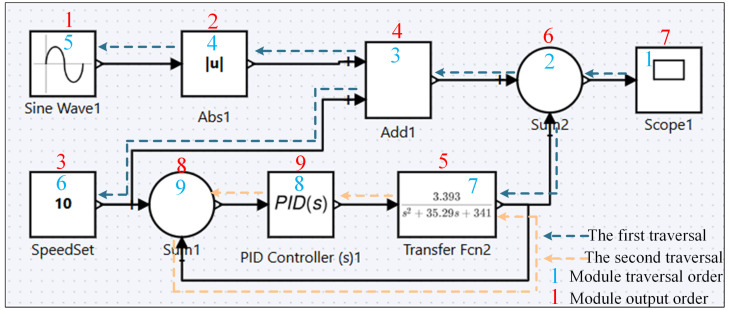
Modules’ output chain design.

A recursive traversal method is used to establish the output links, guaranteeing that the order of module codes aligns with the control logic. As shown in Figure 4, the system starts from terminal modules (such as the Scope module) and recursively traverses backward until it reaches starting modules or previously traversed modules. For modules with multiple input ports, the traversal proceeds sequentially according to the port order.

In Figure 4, the blue labels indicate the traversal order of the modules, while the red labels indicate the arrangement order of the generated code blocks. Through this traversal approach, the server efficiently establishes the output block sequence for all modules, ensuring the accuracy and correctness of the generated code logic.

**Figure 5:**
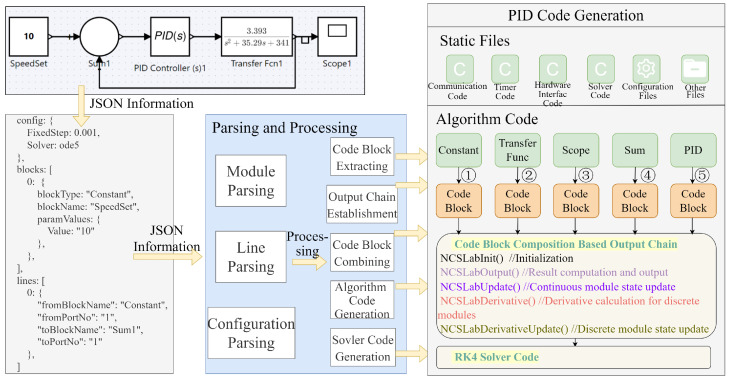
Code generation processes for PID closed-loop control algorithm.

To comprehensively illustrate the automated code generation process, a detailed case of PID closed-loop control algorithm implementation is presented. As shown in Figure 5, users first construct the PID control block diagram through the graphical interface, where each functional module can be double-clicked for parameter tuning. Upon completion, the compilation command packages all diagram information into a JSON format (demonstrated in Figure 5), containing complete module details (including module IDs, name, type, and internal parameters) and connection/line details (including line IDs, source ports, and destination ports). After sending to the server, the system reads and processes the JSON data. The server then configures each module’s C code block according to the extracted parameters and combines the code blocks together in the correct order based on the established output link. Specifically, every module implements certain functions (NCSLabInit(), NCSLabOutput(), NCSLabUpdate(), NCSLabDerivative(), and NCSLabDerivativeUpdate()). These functions are then combined according to the module connections to produce the final complete C code project. For numerical computation during each control cycle, a fourth-order Runge–Kutta (RK4) [23] solver is employed. So far, the automated code generation for PID control is finished.

### 4.2. System-Wide Simulation

Section 4.1 delineates the fundamental workflow and operational principles of the code generation system. While the preceding discussion focuses on individual control block diagrams, an essential question arises: how can system-level swarm control be implemented within this unified framework?

To address this challenge, the system architecture implements subsystems as encapsulated controller algorithms with dual operational modes. When configured as a sub-device, the subsystem is conceptually treated as an independent controller node with UDP-based communication. However, during simulation execution, all subsystems, regardless of their sub-device designation, are integrated into a unified system-level simulation without functional distinction. The critical divergence occurs during compilation: subsystems marked as sub-devices are compiled into standalone executable files, while standard subsystems remain integrated within the main algorithm. This approach maintains simulation consistency while enabling distributed deployment.

**Figure 6:**
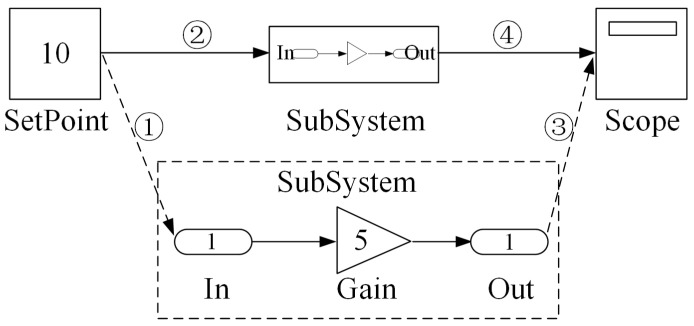
Multi-layer system converted into an equivalent single-layer configuration.

The code generation process for each subsystem is the same as the fundamental principles and workflow described in Section 4.1. However, the introduction of subsystems transforms the algorithm architecture from a single-layer structure to a multi-layer system. To address the complexity of multi-layer traversal, a subsystem virtualization technique is employed that effectively flattens the multi-layer structure into a single-layer structure. As illustrated in Figure 6, this process converts a two-layer system into an equivalent single-layer configuration.

Compared to the basic case presented in Section 4.1 (without subsystems), the modified method performs connection information modification before parsing, where replacing original connections ② and ④ with new connections ① and ③, respectively. This virtualization mechanism conceptually elevates all subsystem modules to the primary (first) layer, while maintaining their logical relationships through the modified connection scheme.

### 4.3. Distributed Deployment

For distributed deployment scenarios, the system compiles multiple subsystem control algorithms from a single control block diagram and subsequently deploys them to respective controllers based on their designated IP addresses and port configurations. When a subsystem module is configured as a sub-device within the block diagram, the compilation process treats it as an independent control algorithm. Specifically, the internal block diagram of each sub-device undergoes autonomous code generation following the same principle and workflow described in Section 4.1.

Concurrently, all remaining algorithm modules in the main block diagram (excluding sub-devices) are collectively compiled into another independent control algorithm. While these sub-devices are visually connected via signal lines in the block diagram, the compilation process automatically establishes UDP-based communication channels between their input/output ports. This abstraction eliminates the need for manual UDP configuration, as the system automatically resolves and implements all necessary inter-device communication relationships.

A critical requirement for sub-device configuration is the specification of IP addresses during module setup. This addressing information enables the system to (i) uniquely identify each sub-device, and (ii) autonomously establish proper communication mappings between distributed components. The complete automation of these networking considerations significantly simplifies the deployment process while ensuring correct distributed operation.

Following the distributed deployment of the multi-vehicle coordination algorithms to their respective controllers, the proposed framework enables centralized real-time monitoring [24]. The system provides customizable monitoring interfaces, allowing users to construct multi-dimensional monitoring dashboards, such as dynamic charts, numerical input boxes, interactive 3D models, etc., for comprehensive swarm state observation. The signal and parameter list are automatically generated based on the control algorithm and its internal parameters, offering two key capabilities, which are flexible signal observation and parameter tuning, where users can selectively monitor any signal of interest and modify real-time parameters.

This section presents the key technical innovations of the proposed UUV swarm control system, focusing on three core capabilities: automated code generation, swarm simulation, and distributed deployment. The developed system features a self-developed compilation engine that eliminates reliance on commercial tools like MATLAB/Simulink, while providing native support for swarm-level simulation and deployment. Specifically, the platform’s automated code generation component translates control algorithms directly into executable code without external dependencies. The integrated simulation environment enables comprehensive validation of swarm behaviors before deployment. Most notably, the system supports distributed implementation across multiple physical controllers through an automated deployment pipeline. This integrated approach addresses critical limitations in current UUV development methodologies by providing a unified workflow from algorithm design to physical implementation, all within a self-sufficient software framework.

## 5. Experimental Setup

This section details the configuration of the experimental platform, including the hardware, software, UUV dynamic model parameters, and the controller gains used for validation. The setup supports both high-fidelity simulation and deployment to physical hardware.

### 5.1. Platform Configuration

The experiments were conducted on the integrated platform described in Section 3. The client layer was accessed via a standard web browser. The server layer operated on a high-performance workstation equipped with an Intel i7-13700K CPU and 64 GB RAM, running the Ubuntu 20.04 LTS operating system. The swarm layer consisted of multiple embedded controllers (Raspberry Pi 4 Model B) for algorithm execution. For the purpose of this case study, the UUV dynamics were simulated within the platform’s digital twin environment to enable controlled and repeatable validation of the core workflow.

### 5.2. System Parameters

The key parameters for the UUV dynamic model and the corresponding controller gains are summarized in Table 2. These parameters are representative of a small-class UUV and were used in the subsequent trajectory tracking case study.

The following section will present the case study and results obtained using this experimental setup.

### 5.3. Inter-Vehicle Communication Implementation

Cooperative control of UUV swarms necessitates inter-vehicle communication, which faces significant constraints in underwater environments. Practical underwater communication, particularly via acoustics, is characterized by low bandwidth, high latency, high bit error rates, and intermittent connectivity.

In this platform, swarm coordination is implemented through a User Datagram Protocol (UDP) network, abstracting the physical layer for controlled experimentation. This setup provides a high-bandwidth, low-latency testing environment ideal for rapid control logic validation. Crucially, the use of UDP inherently introduces packet loss and variable delay. This design choice allows for the development and validation of swarm control algorithms that must be robust to non-ideal communication. The implemented network abstraction layer is designed to facilitate a future transition to in-water testing by replacing the UDP transport with drivers for acoustic modems, while the core swarm algorithms, validated under simulated constraints, remain functionally unchanged.

## 6. Experimental Case Study

This section demonstrates the effectiveness of the proposed framework through comprehensive UUV swarm experiments, showcasing critical functionalities as described in Section 4. Using UUV cluster control as a case study, it systematically validates the platform’s capabilities in simulating complex swarm behaviors in virtual environments, implementing rapid prototype control through automated code generation and deployment, and establishing real-time synchronization between physical systems and their digital twins. The experimental processes and results not only verify the technical feasibility of the integrated approach but also highlight the platform’s unique advantages in bridging the gap between theoretical multi-vehicle coordination algorithms and practical implementations.

### 6.1. Mathematical Model of UUV Motion

For simplicity in modeling, this study considers only the horizontal plane motion of the UUV, neglecting three-dimensional effects. The mathematical modeling of UUV motion is conventionally established through the Earth-fixed coordinate system and the body-fixed coordinate system [25]. The Earth-fixed coordinate system adopts a point on the Earth’s surface as its origin, with the X-axis oriented northward and the Y-axis eastward, forming an X-Y plane tangent to the Earth’s surface. The body-fixed coordinate system originates at the vessel’s center, where the X-axis aligns with the UUV’s longitudinal axis parallel to the sea surface, while the Y-axis extends perpendicularly to the portside from the central longitudinal section, maintaining parallelism with the sea surface. The symbolic definitions employed in the UUV mathematical model are presented in Table 3.

As documented in [25,26], the kinematic model of the UUV can be mathematically formulated as follows:

(1) $$\left\{\begin{array}{c} \dot{x}=u\cos\psi -v\sin\psi \\ \dot{y}=u\sin\psi +v\cos\psi \\ \dot{\psi }=r \end{array}\right..$$

The dynamic models can be expressed as follows:

(2) $$\left\{\begin{array}{c} m_{u}\dot{u}=f_{u}\left(u,v,r\right)+\tau_{u}\\ m_{v}\dot{v}=f_{v}\left(u,v,r\right)\\ m_{r}\dot{r}=f_{r}\left(u,v,r\right)+\tau_{r} \end{array}\right..$$

### 6.2. Trajectory Tracking Control for Underactuated UUVs

**Figure 7:**
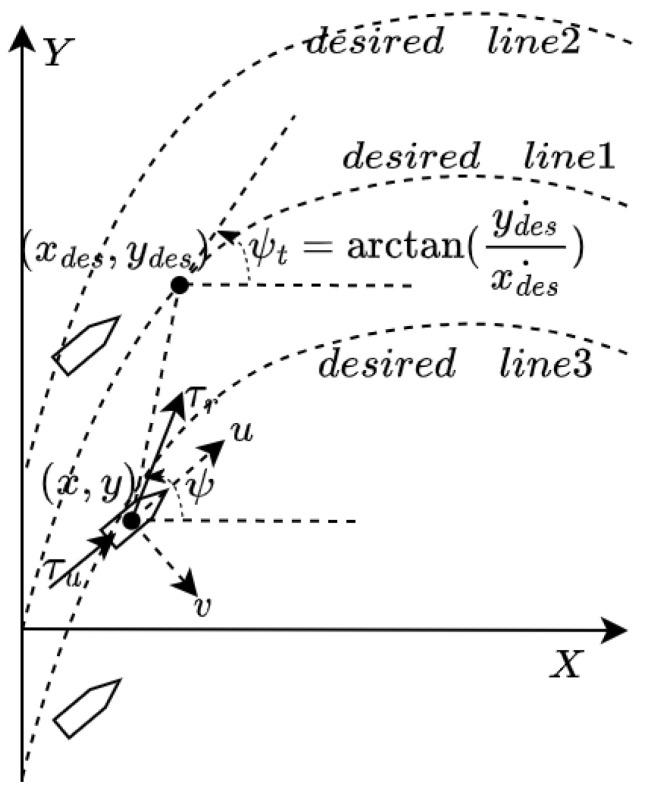
Three-UUV trajectory tracking control.

The underwater vehicle formation uses a leader–follower setup with three vessels, shown in Figure 7. The leader UUV follows a pre-planned path defined by the equations x=2−2cos(0.1t) and y=2sin(0.1t), where *t* is time. This creates a forward-moving sinusoidal trajectory. The two follower UUVs maintain a fixed parallel formation relative to the leader. They track the leader’s real-time position ($x_{leader}$, $y_{leader}$) but stay offset by 2 m on either side. Specifically, the first follower follows ($x_{leader}$, $y_{leader}+2$) while the second follows ($x_{leader}$, $y_{leader}-2$). This arrangement keeps both followers always 2 m above and below the leader’s path respectively, while moving forward at the same speed. The result is a stable three-vehicle formation where all units progress together longitudinally while maintaining constant lateral spacing.

The proposed control framework employs a cascaded PID structure to address the underactuation challenge, where only thrust $\tau_{u}$ and rudder torque $\tau_{r}$ are available for trajectory tracking. The control laws for the UUV are formulated as follows:

(3) $$\left\{\begin{array}{c} \tau_{u}=K_{P,pos}*e_{pos}+K_{I,pos}*\int e_{pos}dt+K_{D,pos}*\dot{e_{pos}},\\ \tau_{r}=K_{P,ang}*e_{ang}+K_{I,ang}*\int e_{ang}dt+K_{D,ang}*\dot{e_{ang}}, \end{array}\right.$$

where the position error $e_{pos}$ and angular error $e_{ang}$ is defined as follows:

(4) $$\left\{\begin{array}{c} e_{pos}=\sqrt{{\left(x_{des}-x\right)}^{2}+{\left(y_{des}-y\right)}^{2}},\\ e_{ang}=\psi_{des}-\psi . \end{array}\right.$$

Here, (x,y) represents the UUV’s current position, and $\left(x_{des},y_{des}\right)$ denotes the desired position at time *t*. ψ is the UUV’s current orientation in the X–Y coordinate system, and $\psi_{des}$ is the desired heading angle computed as follows:

$$\psi_{des}=\frac{1}{2}\left(\arctan\left(\frac{\dot{y_{des}}}{\dot{x_{des}}}\right)+\arctan\left(\frac{y_{des}-y}{x_{des}-x}\right)\right).$$

To ensure stability, the heading loop is tuned aggressively to achieve fast orientation convergence, while position loop gains are carefully balanced to avoid overshoot.

### 6.3. Algorithm for the Integrated UUV Swarm Development Workflow

The core operational workflow of the proposed integrated platform is summarized in Algorithm 1, which provides a high-level procedural description for regenerating the process from swarm control algorithm design to physical deployment and monitoring.
**Algorithm 1** Integrated UUV swarm development workflow.**Require:** 
User-defined swarm control task, Number of UUVs *N***Ensure:** 
Deployed and operational UUV swarm with real-time monitoring  1:**Stage 1: Algorithm Design and System Configuration**  2:Construct the global swarm control algorithm *G* using the graphical interface.  3:Decompose *G* into *N* individual controller algorithms $C_{1},C_{2},\ldots ,C_{N}$, each encapsulated as a *sub-device* module.  4:For each sub-device $C_{i}$, assign a unique controller IP address $IP_{i}$.  5:**Stage 2: System-wide Co-simulation and Validation**  6:Simulate the entire swarm system within the unified virtual environment.  7:Validate collective behaviors, inter-agent interactions, and control logic.  8:Tune parameters of $C_{1},C_{2},\ldots ,C_{N}$ based on simulation results.  9:**if** performance is unsatisfactory **then**10:      **goto** Line 2               ▹ Refine the algorithm design11:**end if**12:**Stage 3: Automated Code Generation & Partitioning**13:For the validated global algorithm *G*, invoke the automated code generation engine.14:The engine compiles *G* and automatically partitions it into N+1 executables: $E_{1},E_{2},\ldots ,E_{N}$ for each sub-device, and $E_{main}$ for the main coordinator (if any).15:The engine automatically resolves and embeds UDP communication protocols between $E_{1},E_{2},\ldots ,E_{N},E_{main}$ based on the signal lines in *G*.16:**Stage 4: Distributed Deployment**17:Deploy each executable $E_{i}$ to its corresponding physical controller at address $IP_{i}$ via the platform’s one-click deployment interface.18:Establish communication links between the controllers and the central monitoring server.19:**Stage 5: Real-time Operation & Monitoring**20:Activate the swarm. Each controller *i* executes its respective algorithm $E_{i}$.21:Initiate real-time data stream from all controllers to the server.22:Visualize swarm kinematics and dynamics in the 3D digital twin interface.23:Monitor key signals and perform online parameter tuning via the customizable dashboard.24:**while** swarm mission is ongoing **do**25:    Continue monitoring and logging.26:**end while**

The following subsection will provide a detailed, step-by-step explanation of this workflow using a concrete UUV formation control case study.

### 6.4. Procedure for the UUV Swarm Control System Design, Simulation and Implementation

**Figure 8:**
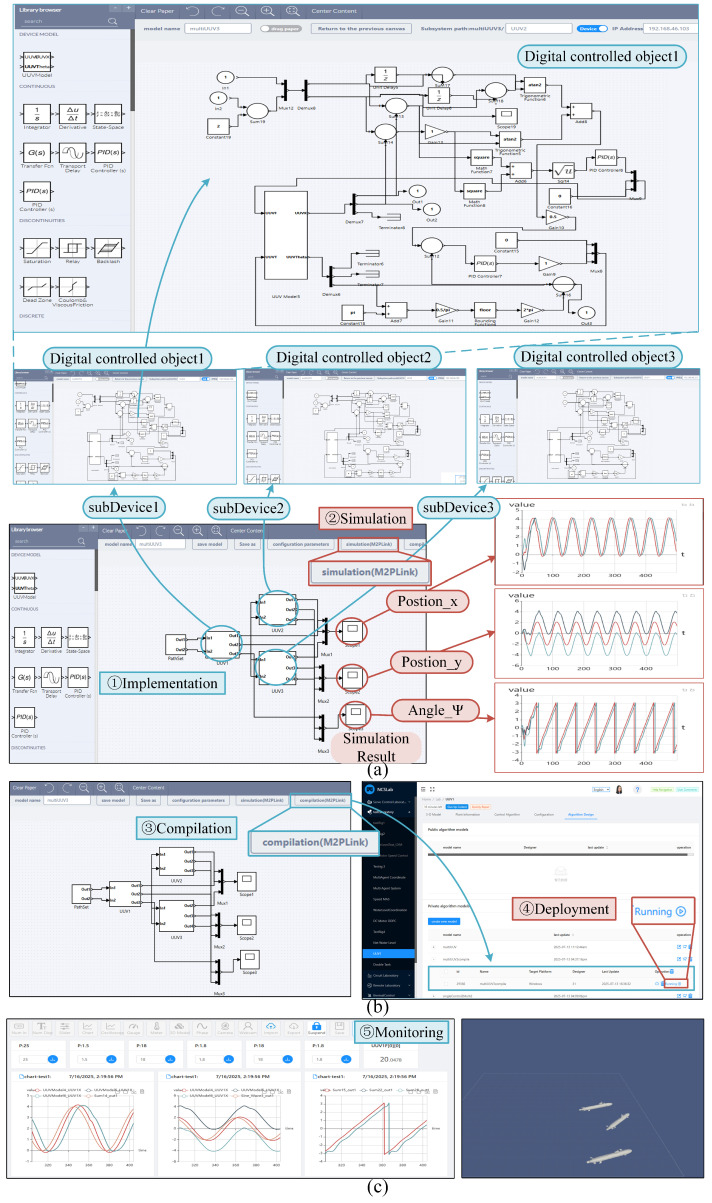
User interfaces of the algorithm design, simulation, compilation, deployment, and monitoring for the UUVs swarm control. (**a**) Design and simulation for UUVs swarm control; (**b**) compilation and deployment for UUVs swarm control; (**c**) monitoring and 3D visualization for UUVs swarm control.

The proposed experimental platform supports the comprehensive implementation of UUV swarm control, encompassing algorithm design, simulation, compilation, deployment, and monitoring. This integrated approach addresses existing methodological limitations by unifying the entire development workflow into a single environment. Here, users can create control algorithms for both individual UUVs and the coordination of the entire group. The operational workflow proceeds as follows:

1. *Algorithm Design and Simulation:* First, users construct control algorithms for three UUVs within the platform’s graphical interface, with each UUV’s control logic implemented as an independent sub-device module. Critical to the deployment process, each sub-device must be configured with a unique IP address to ensure proper algorithm distribution to the designated physical controllers during later stages. Following algorithm development, the platform’s simulation environment allows for immediate validation of control performance and parameter tuning, as demonstrated in Figure 8a.
2. *Compilation and Deployment:* Upon achieving satisfactory control performance through simulation, users initiate the compilation process for the complete coordinated control system (shown in Figure 8b). The platform automatically generates executable binaries for each sub-device, which are then listed in the compilation output panel. Through simple selection and one-click deployment, these compiled algorithms can be synchronously distributed to their corresponding physical controllers via the platform’s automated deployment interface.
3. *Monitoring:* Finally, users can configure customized monitoring interfaces (shown in Figure 8c) for real-time swarm control and visualization. This includes comprehensive 3D visualization capabilities that provide intuitive observation of swarm behaviors, enabling both performance evaluation and parameter adjustment during physical implementation.

Through the experimental validation of UUV multi-vehicle coordination, the proposed framework demonstrates the following key advantages: (i) system-level swarm development automatically generates distributed control code and communication protocols from a single algorithm diagram, significantly improving development efficiency; (ii) the all-in-one architecture seamlessly integrates design, simulation, and deployment in a unified environment, removing dependency on third-party tools; (iii) zero-code programming enables intuitive algorithm development through graphical interfaces, eliminating manual coding requirements. These innovations establish an efficient framework for UUV swarm research and applications.

## 7. Performance and Scalability Analysis

This section provides a comprehensive evaluation of the proposed platform, analyzing both its scalability with increasing swarm size and its computational efficiency on embedded hardware.

### 7.1. Scalability with Swarm Size

The architectural design of the platform inherently supports scalability through several key mechanisms:

1. *Distributed and Parallel Simulation:* Unlike monolithic simulation environments where a single process computes the entire system’s dynamics, our platform leverages a multi-threaded architecture. Specifically, the dynamics of each UUV in the swarm are compiled into an independent executable and simulated in a separate, concurrent thread on the server. This parallelization ensures that the computational load is distributed across available CPU cores.
2. *Decentralized Physical Deployment:* For physical deployment, the control logic is executed on distributed embedded controllers (Raspberry Pi units). This architecture completely decentralizes the real-time computational load. The addition of new UUVs does not impose a processing burden on a central unit, as each vehicle operates autonomously.
3. *Asynchronous Communication Model:* The use of UDP for inter-vehicle communication, both in simulation and deployment, avoids the overhead of connection-oriented protocols, making the network layer lightweight and scalable.

**Figure 9:**
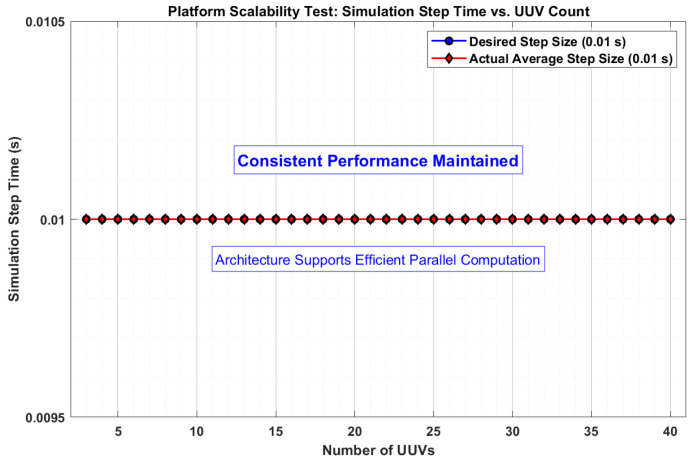
The consistency of the simulation step time (fixed at 0.01s) as the UUV swarm size increases from 3 to 40 vehicles, demonstrating the scalable performance of the parallel simulation architecture.

To quantitatively evaluate scalability, we conducted a simulation stress test, increasing the swarm size from 3 to 40 UUVs while maintaining the same leader–follower formation control task. The critical metric for real-time simulation performance is the ability to maintain a fixed simulation step size. As shown in Figure 9, the platform successfully maintained the hard real-time requirement of a 0.01 s simulation step across all tested swarm sizes. This performance is achieved because the parallel threading model effectively utilizes the multi-core processing resources of the server (described in Section 3), preventing a single simulation thread from becoming a bottleneck. The results demonstrate that the simulation framework scales efficiently within the computational limits of the hardware, without degradation in temporal resolution. This architecture provides a robust foundation for simulating and deploying large-scale swarms.

### 7.2. Computational Performance and Efficiency

Beyond scalability, we evaluated the computational performance and resource consumption of the automatically generated control code on the target embedded hardware (Raspberry Pi 4B with 4 GB RAM). The results, averaged over 1000 control cycles for the PID-based formation controller, are summarized in Table 4. The control algorithm exhibited a deterministic execution profile, with an average execution time per control cycle significantly lower than the 10 ms simulation step, confirming substantial computational headroom for additional tasks. The minimal memory footprint further validates the lightweight nature of the generated code.

### 7.3. Hardware Portability

The platform’s portability was validated by deploying the same compiled control algorithm onto different hardware platforms: a Raspberry Pi 4B and a lower-performance Raspberry Pi 3B+. The code executed correctly on both platforms without modification, demonstrating binary-level portability across ARM architectures. The primary difference was observed in execution time, which increased to an average of 0.28 ms on the Pi 3B+, while memory usage remained consistent. This result confirms that the platform can target heterogeneous hardware, with performance scaling predictably based on device capabilities.

## 8. Conclusions and Future Work

This study has presented an integrated experimental platform for UUV swarm control, successfully addressing the critical gap of fragmented development tools by unifying algorithm design, simulation, and deployment into a cohesive, end-to-end workflow. The proposed system, built on a modular Browser/Server architecture, demonstrated its efficacy through three core capabilities: system-wide co-simulation, automated code generation, and distributed deployment. A case study on leader–follower formation control validated the platform’s ability to significantly accelerate the development cycle. Specifically, the integrated workflow reduced the code development and deployment time by approximately 80% compared to a conventional manual approach, while scalability tests confirmed that a consistent real-time simulation step of 0.01 s is maintained for swarms of up to 40 UUVs.

Looking forward, this platform establishes a foundational framework for advancing UUV swarm technologies. Its integrated workflow is designed to significantly lower the barrier for implementing and testing advanced swarm behaviors in the future. A key direction for our subsequent work will be to leverage this platform to implement and empirically validate more sophisticated control algorithms, including those based on PSO, A*, and other AI-based methods, thereby bridging the gap between theoretical research and practical deployment. Our immediate future work will focus on enhancing the system’s robustness for real-world oceanic environments. This includes the implementation of more complex mission scenarios (obstacle avoidance, adaptive reconfiguration), the development of fault-tolerant mechanisms to ensure operational safety and the design of communication-efficient control algorithms capable of operating effectively in bandwidth-limited underwater acoustic channels. By addressing these critical challenges, we aim to transition the technology from a powerful development tool to a reliable solution for complex marine exploration, defense, and autonomous underwater operations.

## Figures and Tables

**Table 1:** Comparative analysis of multi-agent experimental platforms.

Feature/Platform	Gazebo/Webots	Robotarium	CPSwarm	Our Platform
Unified Graphical Design	Limited	No	✓	✓
System-wide Co-simulation	✓	No	✓	✓
Automated Code Generation	No	No	✓	✓
Distributed Deployment	No	✓(Pre-built)	Limited	✓
UUV-Specific Dynamics	Limited	No	No	✓
Digital Twin Visualization	Limited	No	No	✓
End-to-End Workflow	No	No	Partial	✓

**Table 2:** UUV model and controller parameters.

Parameter Category	Symbol	Value
UUV Vehicle (REMUS 600)		
Mass	*m*	240 kg
Dimensions (L/D)	L/D	4.3 m/0.324 m
Weight/Buoyancy	W=B	2364 N
BG distance	$z_{g}-z_{b}$	0.02 m
Resistance	*r*	0.006
Hydrodynamic Coefficients		
Added mass (x,y,z)	$m_{1},m_{2},m_{3}$	170, 140, 140 kg
Added mass (rotational)	$m_{4},m_{5}$	173, 173 kg·m^2^
Linear damping (surge, sway, heave)	$X_{u},Y_{v},Z_{w}$	−70, −100, −100 N·s/m
Linear damping (rotational)	$K_{p},M_{q},N_{r}$	−50, −50, −50 N·m·s/rad
Quadratic damping (surge, sway, heave)	$X_{uu},Y_{vv},Z_{ww}$	−100, −200, −200 N·s^2^/m^2^
Quadratic damping (rotational)	$K_{pp},M_{qq},N_{rr}$	−100, −100, −100 N·m·s^2^/rad^2^
Controller Gains		
Position proportional gain	$K_{P,pos}$	18.0
Position integral gain	$K_{I,pos}$	0.0
Position derivative gain	$K_{D,pos}$	0.0
Heading proportional gain	$K_{P,ang}$	1.8
Heading integral gain	$K_{I,ang}$	0.0
Heading derivative gain	$K_{D,ang}$	0.0
Actuation Limits		
Max surge thrust	$\tau_{u_{max}}$	50 N
Max yaw moment	$\tau_{r_{max}}$	30 N·m

**Table 3:** Symbol definitions for the UUV mathematical model.

Reference Frame	Symbol	Meaning	Unit
Earth-fixed	*x*	X-coordinate	m
	*y*	Y-coordinate	m
	ψ	Yaw angle	rad
Body-fixed	*u*	Surge velocity	m/s
	*v*	Sway velocity	m/s
	*r*	Yaw rate	rad/s
Control Inputs	$\tau_{u}$	Surge thrust	N
	$\tau_{r}$	Yaw moment	N·m
Inertial Terms	$m_{u}$, $m_{v}$, $m_{r}$	Mass and inertia	kg, kg·m^2^
Hydrodynamic Forces	$f_{u}\left(\cdot \right)$	Coriolis/centripetal, damping	N
	$f_{v}\left(\cdot \right)$	Unmodeled dynamics	N
	$f_{r}\left(\cdot \right)$	Rotational effects	N·m

**Table 4:** Performance metrics of the generated control code.

Performance Metric	Value
Average Execution Time	0.12 ms
Worst-Case Execution Time	0.25 ms
RAM Footprint	4.7 MB
CPU Utilization	∼3%

## Data Availability

The datasets presented in this article are not readily available due to technical limitations. Requests to access the datasets should be directed to authors.
